# Supplementary material for: Prevalence and factors associated with diagnosed diabetes mellitus among Asian Indian adults in the United States
Source: PLOS Glob Public Health. 2023 Feb 2;3(2):e0001551. doi: 10.1371/journal.pgph.0001551 (PMC10021922; doi:10.1371/journal.pgph.0001551)
Supplement: Attachment — Submitted filename: Response to reviewers_12.23.2022.docx [file pgph.0001551.s001.docx]

Ransome Eke, M.D., Ph.D., MPH

Academic Editor

PLOS Global Public Health

We thank the reviewers for their valuable feedback. The reviewer’s comments are *italicized* and our responses to the reviewer’s comments are in blue font.

As per the journal’s requirements we are enclosing the following files:

1. A rebuttal letter that responds to each point raised by the editor and reviewer(s). We have uploaded this letter as a separate file labeled 'Response to Reviewers'
2. A marked-up copy of our manuscript that highlights changes made to the original version. We have uploaded this as a separate file labeled 'Revised Manuscript with Track Changes'.
3. An unmarked version of our revised paper without tracked changes. We have uploaded this as a separate file labeled 'Manuscript'.

Reviewers' comments:

Reviewer #1:

*This study seems like a great initiative to increase awareness about NCD among south Asians.*

Thank you for the positive feedback.

*I have a few concerns here.*

*1. Self reporting of diagnosed DM cases without any investigations will potentially create a bias. Asymptomatic early DM cases have been excluded/missed here. Is there any investigation report in their personal file?*

We acknowledge that self-reports of physician-diagnosed DM cases can create a bias because the self-report may exclude asymptomatic early DM cases. For example, in the NHIS, the sampled adults can indicate diagnosed "borderline diabetes." However, the response categories changed in 2016 to "prediabetes or borderline diabetes. Therefore, to ensure that our diagnosed DM cases are not affected by changes in the response categories in 2016, we included only physician-diagnosed diabetes in our definition.

To address the reviewer's concern, we classified diabetes into three groups: 1) Diagnosed diabetes, 2) Diagnosed prediabetes or borderline diabetes, and 3) no diabetes. Our descriptive analysis indicated no statistically significant differences in prediabetes or borderline diabetes among Asian Indians and Non-Hispanic White (See table below). Despite pooling years, the sample size for Asian Indians was small to analyze separately.

We have also included the following text in the limitation section.

"It has to be noted that our definition of diabetes included only self-reporting of diagnosed diabetes and may under- or overestimate true prevalence of diagnosed diabetes. Some persons may have been classified as not having diabetes when in fact they had undiagnosed diabetes; as much as one-quarter to more than one-third of all diabetes may be undiagnosed (*Menke A, Casagrande S, Geiss L, Cowie CC: Prevalence of and trends in diabetes among adults in the United States, 1988–2012. JAMA 314:1021–1029, 20158*). A prior study [Cowie, Casagrande, and Geiss, 2018: *prevalence and incidence of Type 2 diabetes and prediabetes, Chapter 3*] reported a 2.9% overall prevalence of undiagnosed diabetes in adults ≥20 years, as detected by A1c/FPG and 5% by A1c/FPG/2-hour PG. Variations in undiagnosed diabetes were also reported with the highest prevalence in undiagnosed diabetes among non-Hispanic Asians and lowest in non-Hispanic whites. Furthermore, a prior national study of diabetes among Asian Indians in the US reported a 3.4% undiagnosed diabetes (Misra et al 2009). Hence, the prevalence of diabetes reported in this study may be underreported by 3 to 5%. Another limitation was our inability to differentiate the type of diabetes in the NHIS data; however, the vast majority (90%–95%) of diagnosed diabetes is estimated to be type 2 (*Centers for Disease Control and Prevention: National Diabetes Statistics Report, 2017. Atlanta, GA: Centers for Disease Control and Prevention, U.S. Department of Health and Human Services, 2017*).

|  |  | **Diabetes** | | **Pre- or**  **Borderline**  **Diabetes** | | **No Diabetes** | | **Chisq** | **p-value** |
| --- | --- | --- | --- | --- | --- | --- | --- | --- | --- |
|  |  | **N** | **Wt. %** | **N** | **Wt. %** | **N** | **Wt. %** |  |  |
|  |  |  |  |  |  |  |  | 2.991 | 0.224 |
|  | White | 10,534 | 8.8 | 4,758 | 4.1 | 93,780 | 87.1 |  |  |
|  | Asian Indian | 137 | 7.5 | 74 | 3.8 | 1,775 | 88.7 |  |  |

*2. Family history of DM- an important risk factor has not been adjusted.*

We recognize that a family history of diabetes is a significant risk factor. Unfortunately, information on the family history of diabetes was available only in 2016. We have included this as a limitation in the discussion section.

*3. 60,020 women have been included in the study. Is there any history of GDM? Have you excluded GDM samples from the study? If so ,please mention that.*

GDM is not included because the NHIS question is worded as follows: “Other than during pregnancy, have you EVER been told by a doctor or other health professional that you have diabetes or sugar diabetes?” We have clarified this in the methods section. We have clarified this in the methods section.

*4. How did you confirm high cholesterol?*

High cholesterol was self-reported. Sample adults who answered “yes” to the question “Have you EVER been told by a doctor or other health professional that you had High cholesterol?” were considered to have high cholesterol.
